# Supplementary material for: Differential Analysis of Venous Sinus Diameters: Unveiling Vascular Alterations in Patients with Multiple Sclerosis
Source: Diagnostics (Basel). 2024 Aug 13;14(16):1760. doi: 10.3390/diagnostics14161760 (PMC11353770; doi:10.3390/diagnostics14161760)
Supplement: Supplementary file 1 [file diagnostics-14-01760-s001.zip › diagnostics-3075005-supplementary.pdf]

Supplemental Table S1. Demographic and Clinical Variables in Relation to EDSS Scores and MS Disease Duration

Variables	EDSS <2	EDSS ≥2	p-Value	EDSS <3	EDSS ≥3	p-Value	Disease duration ≤5 years	Disease duration >5 years	p-Value	Disease duration ≤5 years	Disease duration 5-10 years	Disease duration ≥10 years	p-Value	Difference
	n (%) or Mean±SD	n (%) or Mean±SD		n (%) or Mean±SD	n (%) or Mean±SD		n (%) or Mean±SD	n (%) or Mean±SD		n (%) or Mean±SD	n (%) or Mean±SD	n (%) or Mean±SD		
SSS diameter	5.9±0.7	5.8±0.9	0.688	5.9±0.8	5.8±0.8	0.603	5.9±0.8	5.9±0.8	0.985	5.9±0.8	6.2±0.7	5.7±0.9	0.123	
Sinus rectus	5±0.5	5±0.6	0.793	5±0.6	5±0.5	0.465	5.1±0.5	4.9±0.5	0.154	**5.1±0.5**	**5.2±0.6**	**4.8±0.4**	**0.022**	**1-3**
Right TS	5.6±1.9	5.3±1.5	0.455	5.4±1.7	5.5±1.7	0.924	5.5±1.8	5.4±1.6	0.880	5.5±1.8	5.4±1.9	5.5±1.5	0.974	
Left TS	4.6±1.6	4.2±1.8	0.230	4.5±1.5	4.1±2.1	0.345	4.4±1.7	4.5±1.8	0.742	4.4±1.7	4.2±1.7	4.7±1.8	0.620	
Brainstem lesion	15 (39.5)	22 (56.4)	0.208	**22 (40)**	**15 (68.2)**	**0.047**	16 (43.2)	20 (50)	0.715	16 (43.2)	7 (38.9)	13 (59.1)	0.372	
Spinal lesion	30 (78.9)	32 (82.1)	0.955	42 (76.4)	20 (90.9)	0.208	32 (86.5)	30 (75)	0.325	32 (86.5)	14 (77.8)	16 (72.7)	0.411	
OCB			0.363			0.136			1.000				0.400	
Positive	28 (96.6)	30 (88.2)		43 (95.6)	15 (83.3)		30 (90.9)	29 (93.5)		30 (90.9)	15 (100)	14 (87.5)		
Negative	1 (3.4)	4 (11.8)		2 (4.4)	3 (16.7)		3 (9.1)	2 (6.5)		3 (9.1)	0 (0)	2 (12.5)		
IgG Index	1±0.5	0.8±0.2	0.295	1±0.5	0.8±0.3	0.379	1±0.4	0.8±0	0.565	1±0.4	0.8±0	0.8±0	0.851	
Number of bands	10.9±6.2	10±1	0.819	10.6±5.5	11±0	0.946	10.6±5.2	0±0	NA	10.6±5.2	0±0	0±0	NA	
Total number of lesions	16.1±11.3	20.7±13	0.103	16.9±12.6	22.2±11.1	0.081	17.2±13.7	19.3±10.9	0.460	17.2±13.7	15.2±7.8	22.6±12.1	0.120	
Number of contrast-enhancing lesions	0.2±1	0.8±4.1	0.390	0.6±3.5	0.3±1.5	0.659	0.9±4.2	0±0.2	0.204	0.9±4.2	0.1±0.2	0±0	0.403	
Number of perivenular lesions	10.6±7.3	11.7±6.5	0.492	10.3±7.1	13.2±6	0.101	9.7±6.7	12.3±6.6	0.102	**9.7±6.7**	**9.8±5.2**	**14.2±7**	**0.032**	**1-3**
Central extinction	0.2±0.5	0.4±0.9	0.308	0.2±0.5	0.5±1	0.109	0.1±0.3	0.4±0.8	0.057	0.1±0.3	0.4±0.8	0.4±0.9	0.175	

"n" denotes the total number of participants. Percentages are given in parentheses. Mean values are expressed as mean ± standard deviation (SD). $p < 0.05$ indicate the statistical significance. Abbrevations: MS; Multiple sclerosis, OCB; Oligoclonal band, SSS; Superior sagittal sinus, TS; Transverse sinus, EDSS; Expanded disability status scale, IgG; Immunoglobulin G

Supplemental Table S2. Distribution of Demographic and Clinical Findings and Venous Sinus Diameters of the Patient Group

**Variables**	**SSS diameter**		**Sinus rectus**		**Right Transvers sinus**		**Left Transvers Sinus**	
	**Mean±SD**	**p-value**	**Mean±SD**	**p-value**	**Mean±SD**	**p-value**	**Mean±SD**	**p-value**
Gender		0.388		0.237		0.390		0.367
Woman	5.4±0.9		4.5±0.8		5.2±1.5		4.1±1.6	
Male	5.5±0.9		4.7±0.8		5±1.8		3.8±1.7	
OCB		0.481		0.738		0.537		0.68
Positive	5.9±0.8		5.1±0.6		5.5±1.8		4.4±1.6	
Negative	5.6±1.1		5±0.4		5±2		4.7±2.6	
Brainstem lesion		0.271		0.337		0.379		0.173
There is	6±0.8		5.1±0.6		5.6±1.8		4.7±1.7	
None	5.8±0.8		5±0.5		5.3±1.7		4.2±1.6	
Spinal lesion		**0.003**		**0.008**		0.426		0.818
There is	**6±0.8**		**5.1±0.5**		5.5±1.7		4.5±1.6	
None	**5.3±0.6**		**4.7±0.5**		5.1±1.7		4.4±2.1	

Mean values are expressed as mean ± standard deviation (SD).
$p < 0.05$ indicate the statistical significance.
Abbrevations: OCB; Oligoclonal band, SSS; Superior sagittal sinus.
